# Supplementary material for: Ancient role of sulfakinin/cholecystokinin-type signalling in inhibitory regulation of feeding processes revealed in an echinoderm
Source: eLife. 2021 Sep 7;10:e65667. doi: 10.7554/eLife.65667 (PMC8428848; doi:10.7554/eLife.65667)
Supplement: Figure 1—source data 1. [file elife-65667-fig1-data1.docx]

**Figure 1 – source data 1.** Accession numbers for precursors of the neuropeptides shown in the sequence alignment in Figure 1.

| **Precursor/Peptide name** | **Species name** | **Accession number or PubMed reference ID** |
| --- | --- | --- |
| CCK | *Aplysia californica* | XP_005096263.1 |
| SK/CCK | *Asterias rubens* | ALJ99958 |
| NP12 | *Caenorhabditis elegans* | O01970 |
| Cionin | *Ciona intestinalis* | P16240 |
| CCK | *Crassostrea gigas* | EKC26412.1 |
| SK | *Drosophila melanogaster* | P09040 |
| CCK | *Homo sapiens* | P06307 |
| Gastrin | *Homo sapiens* | P01350 |
| SK/CCK | *Ophionotus victoriae* | ASK86241 |
| CCK | *Platynereis dumerilii* | Contig HAMO01025411.1 (transcriptome prediction) |
| SK/CCK | *Strongylocentrotus purpuratus* | PMID: 28878039 (predicted from the genomic scaffold [AAGJ06000007.1](https://www.ncbi.nlm.nih.gov/nuccore/AAGJ06000007)) |
| SK/CCK | *Stichopus horrens* | [HAMZ01045944.1](https://www.ncbi.nlm.nih.gov/nuccore/HAMZ01045944) (transcript) |
| SK/CCK | *Saccoglossus kowalevskii* | XM_002738068.2 |
| SK | *Tribolium castaneum* | D6WP08 |
