# Supplementary material for: Ancient role of sulfakinin/cholecystokinin-type signalling in inhibitory regulation of feeding processes revealed in an echinoderm
Source: eLife. 2021 Sep 7;10:e65667. doi: 10.7554/eLife.65667 (PMC8428848; doi:10.7554/eLife.65667)
Supplement: Figure 2—source data 1. [file elife-65667-fig2-data1.docx]

**Figure 2 – source data 1.** Accession numbers of the receptor sequences used for the phylogenetic tree shown in Figure 2.

| **Receptor** | **Species name** | **Accession number** | **Phylum** | **References (DOI or journal link) for papers reporting experimental identification of neuropeptide ligands for receptors** |
| --- | --- | --- | --- | --- |
| **Acal CCKR2** | *Aplysia californica* | XM_013090996.1 | Lophotrochozoa |  |
| **Ajap SK/CCKR** | *Apostichopus japonicus* | GHCH01030881.1 | Ambulacraria |  |
| **Arub SK/CCKR** | *Asterias rubens* | MW261740 | Ambulacraria | This study |
| **Cbri CKR1** | *Caenorhabditis briggsae* | XP_002640196.1 | Nematoda |  |
| **Cbri CKR2** | *Caenorhabditis briggsae* | XP_002642853.1 | Nematoda |  |
| **Cele CKR1** | *Caenorhabditis elegans* | NP_491918.3 | Nematoda |  |
| **Cele CKR2** | *Caenorhabditis elegans* | ACA81683.1 | Nematoda | 10.1210/en.2007-1772 |
| **Ctel CCKR** | *Capitella teleta* | ELT89517.1 | Lophotrochozoa |  |
| **Cint CioR1** | *Ciona intestinalis* | Q70SX9 | Urochordata | [10.1530/JOE-11-0410](https://doi.org/10.1530/JOE-11-0410) |
| **Cint CioR2** | *Ciona intestinalis* | H7CE69 | Urochordata | [10.1530/JOE-11-0410](https://doi.org/10.1530/JOE-11-0410) |
| **Cgig CCKR1** | *Crassostrea gigas* | MF787221 | Lophotrochozoa | [10.1038/s41598-018-34700-4](https://doi.org/10.1038/s41598-018-34700-4) |
| **Cgig CCKR2** | *Crassostrea gigas* | MF787222 | Lophotrochozoa | [10.1038/s41598-018-34700-4](https://doi.org/10.1038/s41598-018-34700-4) |
| **Drer CCKR1** | *Danio rerio* | XP_697493.2 | Vertebrata |  |
| **Drer CCKR2** | *Danio rerio* | XP_017213239.1 | Vertebrata |  |
| **Dpul SKR** | *Daphnia pulex* | EFX77608.1 | Arthropoda |  |
| **Dmel SKR1** | *Drosophila melanogaster* | NP_001097023.1 | Arthropoda | [10.1006/bbrc.2002.6459](https://doi.org/10.1006/bbrc.2002.6459) |
| **Dmel SKR2** | *Drosophila melanogaster* | NP_001097021.1 | Arthropoda | 10.4161/fly.21534 |
| **Ggal CCKR1** | *Gallus gallus* | BAJ46148.1 | Vertebrata |  |
| **Ggal CCKR2** | *Gallus gallus* | NP_001001742.1 | Vertebrata | [10.1016/S0167-0115(03)00068-5](https://doi.org/10.1016/S0167-0115(03)00068-5) |
| **Gpau CCKR** | *Glossoscolex paulistus* | GBIL01035016.1 | Lophotrochozoa |  |
| **Hsap CCKR1** | *Homo sapiens* | NP_000721.1 | Vertebrata | [10.1006/bbrc.1993.1610](https://doi.org/10.1006/bbrc.1993.1610) |
| **Hsap CCKR2** | *Homo sapiens* | NP_795344.1 | Vertebrata | https://www.jbc.org/content/268/11/8164.long |
| **Lgig CCKR1** | *Lottia gigantea* | XP_009047144.1 | Lophotrochozoa |  |
| **Lgig CCKR2** | *Lottia gigantea* | XP_009047126.1 | Lophotrochozoa |  |
| **Mmus CCKR1** | *Mus musculus* | NP_033957.1 | Vertebrata | https://jpet.aspetjournals.org/content/282/3/1206 |
| **Mmus CCKR2** | *Mus musculus* | NP_031653.1 | Vertebrata | [10.1038/sj.bjp.0702448](https://dx.doi.org/10.1038%2Fsj.bjp.0702448) |
| **Ovic SK/CCKR** | *Ophionotus victoriae* | MW261741 | Ambulacraria |  |
| **Pcau CCKR** | *Priapulus caudatus* | XM_014813624.1 | Priapulida |  |
| **Spur SK/CCKR** | *Strongylocentrotus purpuratus* | XP_782630.3 | Ambulacraria |  |
| **Skow SK/CCKR1** | *Saccoglossus kowalevskii* | XP_006814715.1 | Ambulacraria |  |
| **Skow SK/CCKR2** | *Saccoglossus kowalevskii* | XP_006814705.1 | Ambulacraria |  |
| **Tcas SKR1** | *Tribolium castaneum* | XP_015835017.1 | Arthropoda |  |
| **Tcas SKR2** | *Tribolium castaneum* | XP_972750.1 | Arthropoda |  |
| **Pame SKR1** | *Periplaneta americana* | AAX56942.1 | Arthropoda |  |
|  |  |  |  |  |
| **Hsap OrexinR1** | *Homo sapiens* | NP_001516.2 | Vertebrata |  |
| **Mmus OrexinR1** | *Mus musculus* | NP_945197.2 | Vertebrata |  |
