# Supplementary material for: Ancient role of sulfakinin/cholecystokinin-type signalling in inhibitory regulation of feeding processes revealed in an echinoderm
Source: eLife. 2021 Sep 7;10:e65667. doi: 10.7554/eLife.65667 (PMC8428848; doi:10.7554/eLife.65667)
Supplement: Transparent reporting form [file elife-65667-transrepform1.docx]

***eLife’s* transparent reporting form**

We encourage authors to provide detailed information *within their submission* to facilitate the interpretation and replication of experiments. Authors can upload supporting documentation to indicate the use of appropriate reporting guidelines for health-related research (see [EQUATOR Network](about:blank)), life science research (see the BioSharing Information Resource), or the [ARRIVE guidelines](about:blank) for reporting work involving animal research. Where applicable, authors should refer to any relevant reporting standards documents in this form.

If you have any questions, please consult our Journal Policies and/or contact us: [editorial@elifesciences.org](about:blank).

**Sample-size estimation**

- You should state whether an appropriate sample size was computed when the study was being designed
- You should state the statistical method of sample size computation and any required assumptions
- If no explicit power analysis was used, you should describe how you decided what sample (replicate) size (number) to use

Please outline where this information can be found within the submission (e.g., sections or figure legends), or explain why this information doesn’t apply to your submission:

No explicit power analysis was used. Sample sizes were selected for each experiment type as detailed below:

**1. Receptor assays**

These experiments involved the use of *in vitro* cell-based receptor assays and, in accordance with standard practice for these types of assays, each experiment was performed in duplicate or triplicate (technical replication) and at least four times (biological replication), as stated in the methods section of the paper and in the legends of the relevant figures – Figure 3 and associated supplementary figures/files.

**2. Analysis of ArSK/CCKP expression in *A. rubens* using mRNA *in situ* hybridization and analysis of ArSK/CCK1 and ArSK/CCKR expression in *A. rubens* immunohistochemistry.**

The findings reported in the paper (Figures 4, 5 and 6) are based on analysis of data obtained from at least three animals for each technique, as stated in the methods.

**3. *In vitro* pharmacology**

The findings reported in the paper (Figure 7) are based on analysis of data obtained from 5 - 9 cardiac stomach preparations (dissected from 5 - 9 animals), 8 - 11 tube foot preparations (dissected from 4 - 6 animals) and 20 - 23 apical muscle preparations (dissected from 10 - 12 animals), as detailed in the methods section and in the figure legend for figure 7.

**4. *In vivo* pharmacology – stomach retraction**

The findings reported in the paper (Figure 8) are based on analysis of data obtained from experiments performed on 6 animals injected with ArSK/CCK1, 7 animals injected with ArSK/CCK2, 8 animals injected with ArSK/CCK2(ns). The same animals were first injected with distilled water (control group) and then injected with the peptide (treated group), as detailed in the methods section and in the legend for figure 8.

**5. *In vivo* pharmacology – effects on feeding behaviour**

The findings reported in the paper (Figure 9) are based on analysis of data obtained from experiments performed on 24 animals for testing the effect of ArSK/CCK1 on feeding (13 animals in control group and 11 animals in treated group) and on 38 animals for testing the effect of ArSK/CCK2 on feeding (19 animals in control group and 19 animals in treated group). These sample sizes are detailed in the methods section and in the legend for figure 9.

**Replicates**

- You should report how often each experiment was performed
- You should include a definition of biological versus technical replication
- The data obtained should be provided and sufficient information should be provided to indicate the number of independent biological and/or technical replicates
- If you encountered any outliers, you should describe how these were handled
- Criteria for exclusion/inclusion of data should be clearly stated
- High-throughput sequence data should be uploaded before submission, with a private link for reviewers provided (these are available from both GEO and ArrayExpress)

Please outline where this information can be found within the submission (e.g., sections or figure legends), or explain why this information doesn’t apply to your submission:

Number of replicates were selected for each experiment type as detailed below:

**1. Receptor assays**

These experiments involved the use of *in vitro* cell-based receptor assays and, in accordance with standard practice for these types of assays, each experiment was performed in duplicate or triplicate (technical replication) and at least four times (biological replication), as stated in the methods section of the paper and in the legends of the relevant figures – Figure 3 and associated supplementary figures/files.

**2. Analysis of ArSK/CCKP expression in *A. rubens* using mRNA *in situ* hybridization and analysis of ArSK/CCK1 and ArSK/CCKR expression in *A. rubens* immunohistochemistry.**

The findings reported in the paper (Figures 4, 5 and 6) are based on analysis of data obtained from at least three animals (biological replications) for each technique, as stated in the methods.

**3. *In vitro* pharmacology**

The findings reported in the paper (Figure 7) are based on analysis of data obtained from 5 - 9 cardiac stomach preparations (dissected from 5 - 9 animals), 8 - 11 tube foot preparations (dissected from 4 - 6 animals) and 20 - 23 apical muscle preparations (dissected from 10 - 12 animals), as detailed in the methods section and in the figure legend for figure 7. For experiments on tube foot and apical muscle a maximum of two preparations from the same animal were used.

**4. *In vivo* pharmacology – stomach retraction**

The findings reported in the paper (Figure 8) are based on analysis of data obtained from experiments performed on 6 animals injected with ArSK/CCK1, 7 animals injected with ArSK/CCK2, 8 animals injected with ArCCK2(ns) (biological replications). The same animals were first injected with distilled water (control group) and then injected with the peptide (treated group) as detailed in the methods section and in the legend for figure 8.

**5. *In vivo* pharmacology – effects on feeding behaviour**

The findings reported in the paper (Figure 9) are based on analysis of data obtained from experiments performed on 24 animals for testing the effect of ArSK/CCK1 on feeding (13 animals in control group and 11 animals in treated group; biological replications) and on 38 animals for testing the effect of ArSK/CCK2 on feeding (19 animals in control group and 19 animals in treated group; biological replications). The sample size is detailed in the methods section and in the legend for figure 8. Starfish that were feeding after 24 h were included in the data analysis and any animal in the control or test group that had not fed on a mussel after 24 hours were discarded from data analysis, as detailed in the method section.

**Statistical reporting**

- Statistical analysis methods should be described and justified
- Raw data should be presented in figures whenever informative to do so (typically when N per group is less than 10)
- For each experiment, you should identify the statistical tests used, exact values of N, definitions of center, methods of multiple test correction, and dispersion and precision measures (e.g., mean, median, SD, SEM, confidence intervals; and, for the major substantive results, a measure of effect size (e.g., Pearson's r, Cohen's d)
- Report exact p-values wherever possible alongside the summary statistics and 95% confidence intervals. These should be reported for all key questions and not only when the p-value is less than 0.05.

Please outline where this information can be found within the submission (e.g., sections or figure legends), or explain why this information doesn’t apply to your submission:

**1. *In vitro* pharmacology**

The statistical test performed was 2-way ANOVA with Bonferroni’s multiple comparison test, using the type of substance tested and concentration as independent factors. Apical muscle and tube foot data were transformed to logarithms to obtain a normal distribution and homogeneity of variances. The *in vitro* effects of ArSK/CCK1 and ArSK/CCK2 (1 μM) on cardiac stomach preparations were compared with the *in vitro* effect of NGFFYamide (100 nM) using a two-tailed Student’s t-test. The statistical test performed is stated in the methods section and in Figure 7.

**2. *In vivo* pharmacology – effects on feeding behaviour**

The effect of ArSK/CCK1 on feeding behaviour was analysed by two tailed Mann-Whitney U-test (time to touch and time to enclose) because these data did not follow a normal distribution when analysed using the D’Agostino & Pearson omnibus normality test. The effect of ArSK/CCK2 on feeding behaviour was analysed by two-tailed Student’s t-test (time to touch) or Welch’s unequal variances t-test (time to enclose). Fisher’s exact test was used to analyse the percentage of successful feeding after the first touch for control and treated starfish. The statistical test performed is stated in the method section and in Figure 9.

Statistical analyses were carried out using Prism 6 (GraphPad software, La Jolla, CA, USA) and differences were considered statistically significant at p < 0.05. This information can be found in the methods section.

For each experiment the results are shown graphically as mean ± s.e.m as stated in the method sections and in the figure legends.

(For large datasets, or papers with a very large number of statistical tests, you may upload a single table file with tests, Ns, etc., with reference to sections in the manuscript.)

**Group allocation**

- Indicate how samples were allocated into experimental groups (in the case of clinical studies, please specify allocation to treatment method); if randomization was used, please also state if restricted randomization was applied
- Indicate if masking was used during group allocation, data collection and/or data analysis

Please outline where this information can be found within the submission (e.g., sections or figure legends), or explain why this information doesn’t apply to your submission:

**1. *In vivo* pharmacology – stomach retraction**

Similar sized animals with all 5 arms intact which had been withheld from a food supply for one week were tested. The same animals were first injected with distilled water and then injected with the peptide.

**2. *In vivo* pharmacology – effects on feeding behaviour**

Similar size animals that met the following criteria were used: (i) all 5 arms were intact, (ii) exhibited a normal righting response and (iii) after twenty-four days of starvation, exhibited normal feeding behaviour on a mussel. Individuals were randomly assigned to different groups and experimenters were blind to group assignment.

This information can be found in the methods section.

**Additional data files (“source data”)**

- We encourage you to upload relevant additional data files, such as numerical data that are represented as a graph in a figure, or as a summary table
- Where provided, these should be in the most useful format, and they can be uploaded as “Source data” files linked to a main figure or table
- Include model definition files including the full list of parameters used
- Include code used for data analysis (e.g., R, MatLab)
- Avoid stating that data files are “available upon request”

Please indicate the figures or tables for which source data files have been provided:

**1. Sequence data**

The sequences of the *A. rubens* SK/CCK-type precursor and *A. rubens* SK/CCK type receptor have been deposited in GenBank and the accession numbers are included in the manuscript.

The raw data for the mass spectra shown in Figure 1 – figure supplement 2 are provided in Figure 1 – source data 2.

The accession numbers for the sequences SK/CCK-type neuropeptides from other taxa that were used for comparison with *A. rubens* SK/CCK-type neuropeptides (Figure 1) are provided in Figure 1 – source data 1.

The accession numbers for the sequences SK/CCK-type receptors from other taxa that were used for the phylogenetic analysis of *A. rubens* SK/CCK-type receptor shown in Figure 2 are provided in Figure 2 – source data 1.

**2. Receptor assays**

A graph showing the selectivity of ArSK/CCKR as a receptor for SK/CCK-type peptides is presented in Figure 3 – figure supplement 1.

The data for the graphs shown in Figure 3 and figure 3 – figure supplement 1 are provided in Figure 3 – source data 1.

**3. *In vitro* pharmacology**

The data for the graphs shown in Figure 7 are provided in Figure 7 – source data 1.

**4. *In vivo* pharmacology – stomach retraction**

The data for the graphs shown in Figure 8 are provided in Figure 8 – source data 1.

**5. In vivo pharmacology – effects on feeding behaviour**

The data for the graphs shown in Figure 9 are provided in Figure 9 – source data 1.
